# Interaction of Ruthenium(II)-dipyridophenazine Complexes with CT-DNA: Effects of the Polythioether Ancillary Ligands

**DOI:** 10.1155/MBD.2001.125

**Published:** 2001

**Authors:** Teresa M. Santos, João Madureira, Brian J. Goodfellow, Michael G. B. Drew, Júlio Pedrosa de Jesus, Vitor Félix

**Affiliations:** 1 Department of Chemistry University of Aveiro Campus Universitário de Santiago Aveiro 3810-193 Portugal; 2 Department of Chemistry The University. Whiteknights, Reading RG6 6AD United Kingdom

## Abstract

The complexes [Ru([9]aneS_3_)(dppz)Cl]Cl **1** and [Ru([12]aneS_4_)(dppz)]Cl_2_ **2** ([9]aneS_3_ = 1,4,7- trithiaciclononane and [12]aneS_4_ = 1,4,7,10-tetrathiaciclododecane) were synthesised and fully characterised. These complexes belong to a small family of dipyridophenazine complexes with non-polypyridyl ancillary ligands . Interaction studies of these complexes with CT-DNA (UV/Vis titrations, steady-state emission and thermal denaturation) revealed their high affinity for DNA . Intercalation constants determined by UV/Vis titrations are of the same order of magnitude (10^6^) as other dppz metallointercalators, namely [Ru(II)(bpy)_2_dppz]S^2+^. Differences between **l** and**2** were identified by steady-state emission and thermal denaturation studies . Emission results are in accordance with structural data, which indicate how geometric distortions and different donor and/or acceptor ligand abilities affect luminescence. The possibility of noncovalent interactions between ancillary ligands and nucleobases by van der Waals contacts and H-bridges is discussed . Furthermore, complex **l** undergoes aquation under intra-cellular conditions and an equilibrium with the aquated form **l**' is attained . This behaviour may increase the diversity of available interaction modes.


### INTERACTION OF RUTHENIUM(II)-DIPYRIDOPHENAZINE COMPLEXES WITH CT-DNA: EFFECTS OF THE POLYTHIOETHER ANCILLARY LIGANDS

Teresa M. Santos*[1], João Madureira[1], Brian J. Goodfellow[1], Michael G. B. Drew[2], Júlio Pedrosa de Jesus[1], and Vitor Félix[1]

[1] Department of Chemistry, University of Aveiro.
Campus Universitário de Santiago, 3810-193 Aveiro, Portugal, teresa@dq.ua.pt
[2] Department of Chemistry, The University. Whiteknights, Reading, RG6 6AD, UK

**Abstract**

The complexes [Ru([9]ane$S_3$)(dppz)Cl]Cl **1** and [Ru([12]ane$S_4$)(dppz)]$Cl_2$ **2** ([9]ane$S_3$=1,4,7-trithiaciclononane and [12]ane$S_4$=1,4,7,10-tetrathiaciclododecane) were synthesised and fully characterised. These complexes belong to a small family of dipyridophenazine complexes with non-polypyridyl ancillary ligands. Interaction studies of these complexes with CT-DNA (UV/Vis titrations, steady-state emission and thermal denaturation) revealed their high affinity for DNA. Intercalation constants determined by UV/Vis titrations are of the same order of magnitude ($10^6$) as other dppz metallointercalators, namely $[Ru(II)(bpy)_2dppz]^{2+}$. Differences between **1** and **2** were identified by steady-state emission and thermal denaturation studies. Emission results are in accordance with structural data, which indicate how geometric distortions and different donor and/or acceptor ligand abilities affect luminescence. The possibility of non-covalent interactions between ancillary ligands and nucleobases by van der Waals contacts and H-bridges is discussed. Furthermore, complex **1** undergoes aquation under intra-cellular conditions and an equilibrium with the aquated form **1'** is attained. This behaviour may increase the diversity of available interaction modes.

### Introduction

The affinity of metal complexes with DNA is normally investigated by determining their intercalative properties. Complexes with dppz (dipyrido[3,2-a:2',3'-c]phenazine) as a ligand, show strong intercalation with DNA due to the extended aromatic heterocyclic surface that extends well outside the central core of the complex, thus minimising steric hindrance between ancillary ligands and DNA. Ruthenium(II)-dppz complexes with polypyridyl ancillary ligands have shown remarkable B-DNA affinity, with binding constants $>10^6$ [1] but they possess low specificity [2].

DNA binding is normally determined by UV/Visible spectroscopy, however luminescence can also be used. $[Ru(bpy)_2dppz]^{2+}$ and other Ru(II)-dppz complexes luminesce in organic solvents, but this emission is absent in aqueous solutions since water molecules deactivate the excited state through hydrogen bonding to the phenazine nitrogen atoms. After DNA addition these complexes show luminescence due to the protection provided by the nucleobases, via intercalation, against solvent quenching. This phenomenon, generally designated as a "molecular light switch", is an indication of an intercalative mechanism [1,3-6]. In contrast to dppz complexes, Ru(II) or Rh(III) phi complexes show no luminescence (phi = 9,10-phenanthrenoquinone diimine) [7].

A number of metallointercalators with high DNA affinity are known [8-12] and more recently efforts have turned toward a search for an increase in the specificity of the metal complex-DNA interaction *via* modification of the ancillary ligands. Two approaches have been used: shape selection and the promotion of favourable ancillary ligand-DNA interactions [13]. Both strategies have been mainly developed for the ligand phi and its derivatives. Shape selection involves, for instance, the use of large ancillary ligands which hinder binding in the minor groove of DNA [14-17]. Our group is interested in the second approach; the design of metal complexes containing ancillary ligands with favourable stabilising interaction with DNA.

There are a few examples where this strategy has been followed. In the early 90's Barton and co-workers [10,18] studied the $[Rh(L)(phi)]^{3+}$ complexes (L = $(NH_3)_4$, $(en)_2$, [12]ane$N_4$ and [12]ane$S_4$). The presence of the axial amines resulted in a specific interaction at 5'-GC-3' sites, as models indicated the possibility of hydrogen bonds between them and $O_6$-guanine. The complex $\Lambda$-$[Rh(en)_2(phi)]^{3+}$ also showed preference for 5'-TX-3' sites, attributed to van der Waals interactions between the methylene chains and the thymine methyl group. In contrast, for the $\Delta$ enantiomer, no specificity was seen. This was also the case for $[Rh([12]aneN_4)(phi)]^{3+}$ where the small $N_{ax}$-Rh-$N_{ax}$ angle ($\approx 160°$) reduced the possibility for specific interactions between the nitrogens and the nucleobases [10,19]. On the other hand, the complex $[Rh([12]aneS_4)(phi)]^{3+}$ shows preference for 5'-AXY-3' sites but how it interacts is still unknown [10,18]. The complex Δ-α-$[Rh\{(R,R)Me_2trien\}(phi)]^{3+}$ was then synthesised in order to prove these hypothesis. The complex showed a remarkably high affinity for DNA (binding constant ≈ $10^8$) and targeted a 5'-TGCA-3' sequence in the major groove. This was confirmed by NMR [20] and further by the single crystal x-ray determination of this metallointercalator with a DNA oligonucleotide [21].

Following our aim of understanding the rules governing interactions between polythioether complexes and DNA we have recently obtained the first polythioether complex with dppz $[Ru([9]aneS_3)(dppz)Cl]PF_6$. In a previous study the packing diagrams of several crystal structures of $[Ru([9]aneS_3)(N\text{-}N)Cl]^+$ complexes (N-N = bidentate polypyridyl) have clearly revealed the presence of hydrogen bonds between ligands suggesting that the same type of H-bonding between DNA nucleobases and the complexes may be possible [22].

In this paper we present the characterisation and the interaction of $[Ru([9]aneS_3)(dppz)Cl]Cl$ **1** and the new complex $[Ru([12]aneS_4)(dppz)]Cl_2$ **2** with DNA. Complex **1** was found to undergo aquation (**1'**) opening up the possibility of further H-bonding [23]. In Ru(II) or Rh(III) saturated polypyridyl / polyamine-polypyridyl mixed systems normally used as metallointercalators, this opportunity is unavailable.

### Experimental

***Reagents:*** $RuCl_3{\cdot}nH_2O$, the polythioether macrocycle ligands $[9]aneS_3$ (1,4,7-trithiacyclonone) and $[12]aneS_4$ (1,4,7,10-tetrathiacyclodecane), 2,2'-bipyridine, 1,10-phenantroline, $(t\text{-But})_4PF_6$ and all solvents (Aldrich), $NH_4PF_6$, $KH_2PO_4$, $K_2HPO_4{\cdot}3H_2O$, NaCl (Merck), Trizma base and Trizma-hydrochloride (Sigma), were of analytical purity and used as received without any further purification. All buffers and solutions for DNA interaction studies were prepared with ultra pure water.

***Instrumentation: Elemental analyses*** were obtained on a LECO CHNS-932 Elemental Analyser. ***ES-MS***: mass spectra were acquired with a VG AutoSpecQ (VG Analytical Manchester, UK) working in an EBE geometry mode, equipped with a VG electrospray source and a syringe pump Phoenix 20CU (Fisons Instruments). A mixture of methanol/water (50:50) was employed as the eluent. The applied accelerating voltage was 4 kV. The electrospray parameters were optimised in order to obtain good signal-to-noise ratios for the ions of interest, the average needle voltage was 2.5 kV and the average sampling cone voltage 50 V. The mass spectrometer was operated at a nominal mass resolution of 1000 (10% valley). Spectra were obtained at a scan rate of 8 s $decade^{-1}$. ***IR*** spectra were run (KBr pellets) on a FTIR Mattson 7000 infrared spectrophotometer and ***$^1H$ NMR*** spectra on either a Bruker DRX500 or AMX300, using 16k data points and a sweep width of 14.04 ppm. Deuterated methanol, acetonitrile, water and dimethylsulphoxide were used as solvents with chemical shifts being referenced to $CD_2HOD$ (δ3.35), $CD_2HCN$ (δ 1.94), HOD (δ 4.75) and dmso (δ 2.50). ***UV/Vis*** spectra and DNA spectrophotometric interaction studies were recorded at room temperature on a Jasco V-560 spectrophotometer. Denaturation and aquation reactions were carried out using peltier temperature controller. ***Steady-state emission*** studies were performed at room temperature on a Spex F111 Fluorolog, with a 150W Xenon lamp and a single monochromator, for both excitation and emission, with no intensity correction. ***Cyclic voltammetric*** characterisation of the complexes was carried out on a BAS CV-50W-1000 Voltammetric Analyser. CV experiments were performed in fresh HPLC quality $CH_3CN$ solutions, in a C-2 glass cell BAS-MF-1082, under $N_2$ at room temperature with $(t\text{-But})_4PF_6$ (0.1 M) as the supporting electrolyte. The reference electrode, a $Ag/Ag^+$ couple, consisted of a $CH_3CN$ solution of $AgNO_3$ in contact with a silver wire placed in glass tubing with a Vycor frit at one end to allow ion transport. Calibration was carried out against a ferrocene solution (1mM). The $Fc/Fc^+$ couple vs $Ag/Ag^+$ showed a ΔEp of 66 mV and $E_{1/2}$ of 89 mV. The working electrode was of vitreous carbon and the auxiliary electrode was a Pt wire (Bas-MW-1032). All the potential data included in the text, even from literature, are in SSC form. Different scan rates and convolution/deconvolution techniques were used in order to obtain consistent results and to optimise the definition and the reversibility of the waves. Oxidation couples that are not chemically reversible are referred to their $E_p$ values. ***Crystallographic Measurements / Structure analysis and refinement***: The X-ray data for complex **2** were collected on a MAR research plate system using graphite Mo-Kα radiation at Reading University (U.K.). The structure was solved by direct methods and refined on F2 using the SHELXS and SHELX within SHELX97 package [24].

***Synthesis of ligands and complexes:***

***a) Synthesis of dppz and the $Ru^{II}$ precursor complexes:*** **dppz** was synthesized according to the literature with slight experimental modifications [25]. Ligand purity was checked by $^1$H-NMR (dmso-$d_6$) [26] and elemental analysis. $\mathbf{[Ru([9]aneS_3)(dmso)Cl_2]}$ [22,27] and $\mathbf{[Ru([12]aneS_4)(dmso)Cl]Cl}$ [28] were obtained as previously described.

***b) Synthesis of the $Ru^{II}$/dppz complexes:*** $\mathbf{[Ru([9]aneS_3)dppzCl]Cl.2.5H_2O}$ **(1)** The synthesis of this compound was based on a previously described procedure [22]. The process was altered slightly in order to avoid contamination with unreacted starting material (507.7mg; 80%). Found: C, 42.1; H, 4.2; N, 8.3; S, 14.5. Calc. for $C_{24}H_{22}Cl_2N_4RuS_3{\cdot}2.5H_2O$: C, 42.4; H, 4.0; N, 8.2; S, 14.2 (*See Discussion*). $\mathbf{[Ru([12]aneS_4)(dppz)]Cl_2{\cdot}5.5H_2O}$ **(2)** $[Ru([12]aneS_4)(dmso)Cl]Cl$ (245.3 mg; 0.5 mmole) and dppz (141.5

mg; 0.5 mmole) were dissolved in 15 ml of degassed absolute ethanol. The resulting solution was refluxed at 85°C for 12 h turning a deep red-orange colour. Standing at room temperature for 2 days, resulted in the formation of a red orange powder which was filtered, washed with cold ethanol (96%), diethyl-ether and then dried at 65°C (313.6 mg; 79%). Crystals suitable for single crystal X-ray determination were obtained. Found: C, 39.1; H, 4.6; N, 7.1; S, 16.2. Calc. for $C_{26}H_{26}Cl_2N_4RuS_4.5.5H_2O$: C, 39.3; H, 4.7; N, 7.1; S, 16.2. **$[Ru(bpy)_2dppz](PF_6)_2$ (3)** was synthesised with slight modifications from the literature [26]. Its purity was checked by $^1$H-NMR ($CD_3CN$) and elemental analysis (Found: C, 46.6; H, 2.8; N, 11.1. Calc. for $C_{38}H_{26}F_{12}N_8P_2Ru$: C, 46.3; H, 2.7; N, 11.4).

***Aqueous solution behaviour of $Ru^{II}$/dppz complexes:*** Prior to the DNA binding studies the solution stability of the complexes was tested. UV/Vis spectra of aqueous phosphate (0.01 M) buffer solutions of the complexes were acquired immediately after the solutions were prepared, within short time intervals and up to 1 or 2 days. Additionally for complex **1** the influence of [$Cl^-$] on its aqueous solution behaviour was also studied by UV/Vis and $^1$H-NMR. UV/Vis spectra (50.μM in complex) were recorded in a 5mM Tris and 20 mM NaCl solution, except for complex $[Ru([9]aneS_3)dppz(H_2O)]^+$, **1'**, where only ultra-pure water was used. Aquation studies were carried out at 37°C, pH 7.4, in 5mM Tris and with variable concentrations of NaCl.

***Binding Studies with Calf Thymus DNA (CT-DNA):*** CT-DNA concentrations were determined by spectrophotometry using $\varepsilon_{260}$ = 6600 $M^{-1}cm^{-1}$ [29]. A $A_{260}/A_{280}$ value of 1.8-1.9 was always observed, indicating that the DNA was free from protein contamination [30].

***a) Spectrophotometric titrations:*** CT-DNA spectrophotometric titrations were carried out at room temperature in 10 mM phosphate buffer pH=7.2. Successive small aliquots, normally 25 μL, of CT-DNA (concentration range from 350-1350 μM) were added to the complex (*ca* 50 μM or 100 μM). Absorbance changes were followed at 414 nm for **1'**, at 412 nm for **2** and at 445 nm for **3**.

***b) Luminescence studies:*** Both CT-DNA and the complexes were prepared in 5 mM Tris buffer and 50 mM NaCl, pH = 7.4. Prior to the experiments, all the solutions were air saturated in order to maintain the quenching level. The complexes (*ca* 60 μM) were excited at their MLCT bands and at the "double-hump" wavelengths [31] in order to choose the best one for emission purposes and then diluted to 6μM for the DNA interaction studies. A baseline correction was performed at 800 nm, allowing better deconvolution of the spectra.

***c) Thermal denaturation studies:*** Thermal denaturation of DNA and of DNA with Ru(II)-complexes were performed by monitoring the absorbance of the samples at 260 nm. The absorbance data were normalised against room temperature values over the whole temperature range. Contributions from the dissociation of the Ru(II)-complexes at this wavelength were not considered over the course of the melting transitions (verified to be very small). The samples were heated till the desired temperature and maintained at equilibrium for 10 minutes before reading the absorbance value. For temperatures between room temperature and 70°C a Jasco V-560 spectrophotometer equipped with a Peltier heating system was used and for temperatures higher than 70°C an external oil bath was utilised. All solutions were prepared with a 10 mM phosphate buffer at pH 7.2, containing 67.5 or 125 μM CT-DNA with [DNA]/[complex] ratios of 5:1, 10:1 or 20:1. $[Ru([9]aneS_3)(phen)Cl]Cl$ [32] was also studied at a [DNA]/[complex] ratio of 5:1, for the sake of comparison. Melting curves were constructed by plotting the relative absorbance (normalised against the value at room temperature) versus temperature [33].

### Results and Discussion

***Method of synthesis:*** The presence of a small amount of water during the synthetic procedure of complex **1** is needed to assure the complete dissolution of the $[Ru([9]aneS_3)(dmso)Cl_2]$ precursor at reflux conditions. Nevertheless, a water/ethanol balance must be attained in order to assure solubility of the dppz ligand or to avoid oxidation problems with the metal centre. Unreacted precursor, which is a source of contamination in the case of complex **1**, is removed by dissolution in water and subsequent slow filtration (48 h) followed by selective precipitation with NaCl. Finally the solid was recrystallised from boiling methanol.

***Characterisation of the complexes:*** The characterisation of complexes **1** and **2** is summarised in Table 1.

***IR***: The characteristic IR bands for the type of macrocycle and polypyridylic ligand present in these complexes appear as expected. The main Ru-S stretching band is located at *ca* 430 $cm^{-1}$ for both complexes. A very weak band corresponding to the Ru-Cl stretching vibration (282 $cm^{-1}$) is seen for the $[9]aneS_3$ complex [22].

***UV/Vis***: The spectra of complexes **1**, **1'** (where $Cl^-$ has been replaced by a coordinated water molecule) and **2**, are shown in Fig.1. The deconvolution of the spectra which has been made by different methods [35] is also presented in the same figure. The electronic absorption spectra of complexes **1-3** show a less energetic band at 400-450 nm usually assigned to MLCT transitions [36], intra-ligand transitions, namely the "double-humped" dppz centred band [31] are also observed in the 380-330 nm range and below 300 nm. Between 300 and 320 nm a shoulder is also seen.

**Table 1.** Characterisation of $[Ru([9]aneS_3)(dppz)Cl]Cl{\cdot}2.5H_2O$ **1** and $[Ru([12]aneS_4)dppz)]Cl_2{\cdot}5.5H_2O$ **2**.

| **Complex →** **Technique ↓** | **$[Ru([9]aneS_3)(dppz)Cl]Cl{\cdot}2.5H_2O$ (1)** | **$[Ru([12]aneS_4)dppz)]Cl_2{\cdot}5.5H_2O$ (2)** |
|---|---|---|
| **IR** ($cm^{-1}$) | $\nu_{Ru\text{-}S}$ (428w), $\nu_{Ru\text{-}Cl}$ (282 vw) [a] | $\nu_{Ru\text{-}S}$ (430 w) |
| **UV/Vis** ($\lambda_{max}$, nm) ($\varepsilon \times 10^{-3}$ $M^{-1}$ $cm^{-1}$) | **1**: 276 (54.2), 315 (3.1) sh, 358 (13.6), 418 (5.3) [b] | 275 (52.9), 307 (22.1) sh, 360(13.5), 367 (13.4), 408 (7.6) [b] |
| | **1'**: 205 (44.8), 276 (55.8), 316 (13.8)sh, 357 (14.0), 414 (5.9)sh [c] | 207 (48.7) , 277 (47.0), 308 (19.4)sh, 361(11.9), 369 (12.1), 412 (6.1) [c] |
| **$^1$H NMR** ($\delta_H$, ppm) | *dppz* - 9.61 (2H,dd), 9.50 (2H,dd), 8.27 (2H,m), 8.09 (2H,m), 7.99 (2H,m) *[9]aneS$_3$* - [3.25-3.00] (4H,m), [3.00-2.65] (8H,m) [a, d] | *dppz* - 9.59 (2H,br), 8.74 (2H,d), [7.69-7.55] (6H,m) *[12]aneS$_4$* - [3.95-3.50] (8H,br), [3.20-2.85] (8H,br) [e] |
| **ES-MS** (m/z) [f] | 599 $[M]^+$ [g] | 659 $[M+Cl]^+$; 312 $[M]^{2+}$ |
| **CV** [h] ($E_{1/2}$, $E_p$:V; $E_p$:mV) | +1.269 ($\Delta E_p$=66), ≈ +1.090 (–) -0.922 ($\Delta E_p$=62), -1.590 (–) | +1.530 (–), +1.146 (–) -0.878 ($\Delta E_p$=62), -1.436 (–) |

[a] Ref. [22]; [b] 5mM Tris, 20 mM NaCl; [c] phosphate buffer 10 mM, pH 7.2; [d] $CD_3OD$; [e] $D_2O$; [f] mono-isotopic masses / $^{102}$Ru isotope; [g] Ref. [34]; [h] in $CH_3CN$ *vs* SSC at a 50 mV/s scan rate.

***$^1$H-NMR:*** The aromatic region of $[Ru([12]aneS_4)dppz)]Cl_2.5.5H_2O$ indicated that a symmetric complex had been formed. Integration confirmed the 1:1 stoichiometry for dppz : $[12]aneS_4$. Broad lines were seen for the $CH_2$ protons of the $[12]aneS_4$ ring and for the $H^3$, $H^6$ protons of dppz (closest to the $[12]aneS_4$ ring) which are most probably be due to rapid chemical exchange relieving strain at the Ru centre [37]. The spectrum of $[Ru([9]aneS_3)dppz)Cl](PF_6)$ has already been discussed [22].

***ES-MS***: Electrospray mass spectrometry allowed the clear identification of the $[M]^+$ ion for complex **1** and the species $[M+Cl]^+$ and $[M]^{2+}$ for complex **2**. The mass spectrum of complex **1** presents the characteristic fragmentation pattern of this type of complex [34]. For complex **2,** the mass spectrum shows mainly ions due to the fragmentation of the crown thioether [38].

***Cyclic Voltammetry:*** Complex **1** shows a reversible one-electron redox wave ($E_{1/2}$=+1.269 V), normally assigned to the $Ru^{3+}/Ru^{2+}$ couple [36], which is seen for other $Ru([9]aneS_3)Cl$/polypyridyls (*eg*, +1.254V for $[Ru([9]aneS_3)(4,4'\text{-}bpy)_2Cl]^+$); +1.243 V for $[Ru([9]aneS_3)(py)_2Cl]^+$) [39] and for other Ru(II)/dppz complexes (+1.29V: $[Ru(bpy)_2dppz]^{2+}$ [26 ]; +1.33V: $[Ru(dppz)_3]^{2+}$) [40]. It appears that the $([9]aneS_3)Cl$ fragment exerts a similar electronic effect on the Ru(II) centre as do two polypiridyls. For complex **2** $E_{pa}(Ru^{3+}/Ru^{2+})$ is +1.530V, which is analogous to other $Ru([12]aneS_4)$/polypridyls [28].

The presence of a Cl $\pi$-donor ligand in complex **1** reinforces the charge density at the metal centre. Thus the Ru-S bond is stabilised by $\pi$-back donation resulting in a more energetically accessible Ru(II) state [28,39,41]. In complex **2**, which has a longer average $Ru\text{-}S_{ax}$ bond length (2.39 Å) than expected for Ru(II)-polythioether complexes with sulfur atoms in *trans* positions (2.32-2.34 Å) [42-46], these $\sigma$-bonds are weaker. Consequently, electronic density on the metal centre drops, diminishing the $\pi$-back bonding ability. Both reasons may explain the $E_{1/2}$ $(Ru^{3+}/Ru^{2+})$ cathodic shift of *ca* 0.26V in complex **1** when compared to **2**. This shift can not be correlated with differences in the macrocyclic ligands, as it is known that chelate ring strain has a relatively small effect on the $E_{1/2}$ values of these $Ru^{3+}/Ru^{2+}$ couples [41,47].

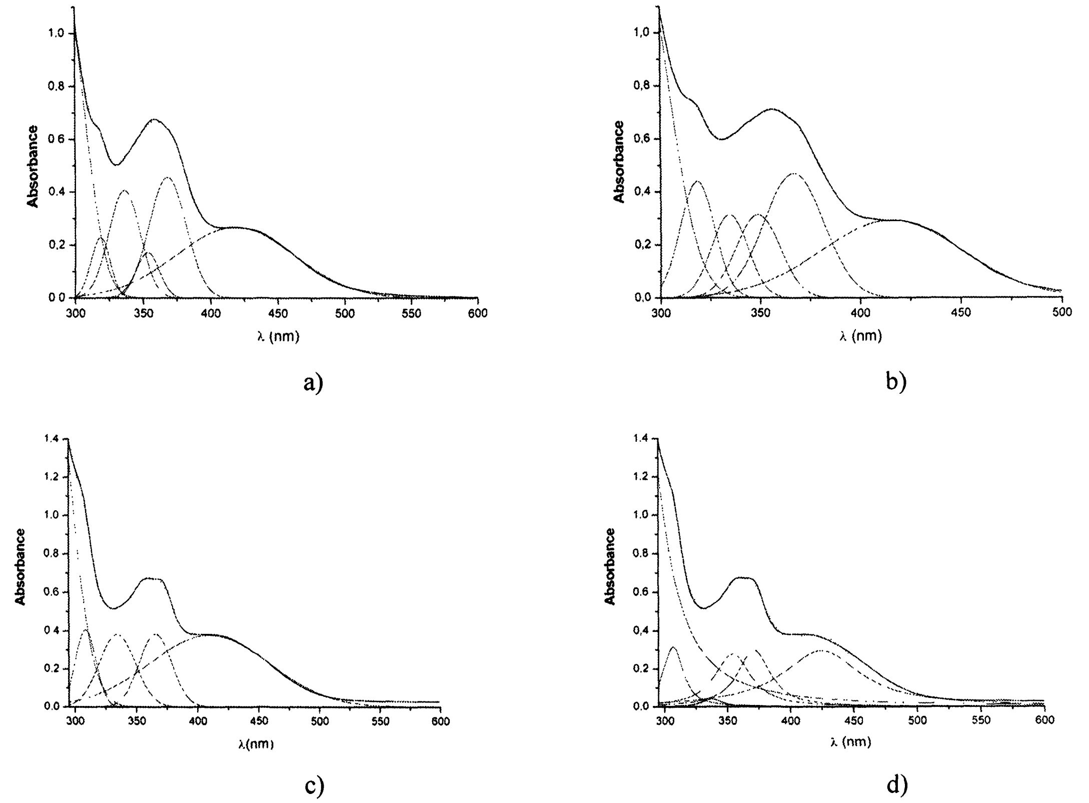

**Fig. 1.** UV/Vis spectra of the 50 μM solution of: a) $[Ru([9]aneS_3)(dppz)Cl]^+$ **1**; b ) $[Ru([9]aneS_3)(dppz)(H_2O)]^{2+}$ **1'**; c) $[Ru([12]aneS_4)(dppz)]^{2+}$ **2** and d) $[Ru([12]aneS_4)(dppz)]^{2+}$ **2**. A Gaussian deconvolution was used for a-c and a Lorentzian for d. The buffer used for **1** and **2** was 5 mM Tris, 50 mM NaCl and for **1'** ultra-pure water.

In the negative potential zone a typical polypyridyl ligand centred redox wave (*ca* –0.9V) is seen for complexes **1**, **2** and **3** [26,31]. This reduction has been identified as a phenazine centred $\pi^*$ orbital type and not as a bpy centred type [26,31,48], as bpy reduction occurs at a much lower potential (*eg* in $[Ru(bpy)_3]^{2+} \approx -1.40V$) [36].

***X-ray diffraction studies:*** The crystals obtained for complex **1** show rough shapes and crumble easily suggesting low mosaicity. Several crystals were investigated and all of them displayed poor diffraction patterns indicating the solid was composed of *ca* 50% crystal and 50% powder. However a crystal structure was determined. After many trial refinements the best model included the hydrogen atoms at calculated positions, anisotropic thermal parameters for ruthenium and sulfur atoms and isotropic parameters for carbon and nitrogen atoms. A high final *R* value of 0.1582 for 1873 reflections with $I > 2\sigma(I)$ was obtained. The final quality of this X-ray structure is not good enough for publication, but the overall geometry of complex is established unequivocally. The molecular structure of $[(Ru([12]aneS_4)dppz)]^{2+}$ cation is shown in Fig. 2 together with selected bond distances and angles subtended at the ruthenium centre. The complex cation exhibits a distorted octahedral co-ordination environment with the equatorial co-ordination plane defined by two nitrogen atoms from the dppz ligand and two macrocyclic sulfur atoms. Six-co-ordination is completed with two remaining sulfur donor atoms of the macrocyclic ligand.

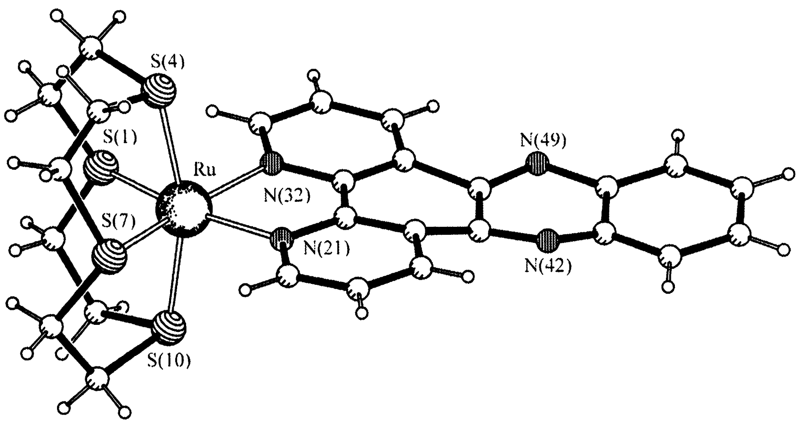

**Fig. 2.** Molecular structure of [(Ru([12]ane$S_4$)dppz)]$^{2+}$ [49]. Selected bond distances [Å] and angles [°]:Ru-N(21) 2.115(15); Ru-N(32) 2.151(19); Ru-S(1) 2.327(9); Ru-S(4) 2.414(9); Ru-S(7) 2.268(11); Ru-S(10) 2.373(9); N(21)-Ru-N(32) 77.2(8), S(10)-Ru-S(4) 162.5(3).

The standard deviations associated with molecular dimensions listed in Fig. 2 indicate they have enough accuracy to characterise the ruthenium co-ordination sphere and their values agree well with those found for other related Ru(II)[12]ane$S_4$-polypyridyl derivatives [28]. The N-Ru-N angles of 77.2(8)°, in the equatorial plane, and the S(4)-Ru-S(10) angle of 162.5(3)°, in the axial plane, are far from their ideal octahedral values. The small bite angle of dppz and the small cavity size of [12]ane$S_4$ result in the non-ideal values [50]. In comparison the crystal structure of [Ru([9]ane$S_3$)(dppz)Cl]$PF_6$ has $S_{ax}$-Ru-Cl angles of 179.0(2)° and 176.5(2)°, in the axial plane, for the two independent cations [22].

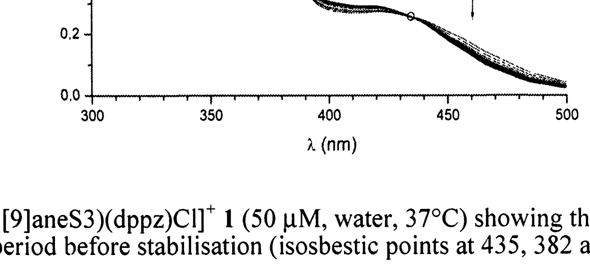

**Fig. 3.** UV/Vis of [Ru([9]aneS3)(dppz)Cl]$^+$ **1** (50 μM, water, 37°C) showing the spectral evolution over a 20 min period before stabilisation (isosbestic points at 435, 382 and 364 nm).

***Behaviour in aqueous solution:*** [Ru([12]ane$S_4$)(dppz)]$^{2+}$ **2** and [Ru(bpy)$_2$(dppz)]$^{2+}$ **3**, in phosphate buffer (10 mM), at room temperature, maintain all spectrophotometric characteristics (intensity and band positions) over a two day period and the presence of $Cl^-$ (20 mM NaCl) only causes slight differences in the intensity of the bands and minor changes in the MLCT band (no labile sites for ligand exchange). Complex **1**, on the other hand, shows clear hydrolytic behaviour under low chloride concentrations, forming the species $[Ru([9]aneS_3)(dppz)(H_2O)]^{2+}$ **1'** (See Fig. 3). Parallel NMR experiments have also demonstrated that aquation is taking place for complex **1** [51].

***Aquation study of [Ru([9]aneS₃)(dppz)Cl]Cl***: In order to study the interactions between complex **1** and DNA in aqueous solutions it is very important to establish the exact degree of aquation at a specific chloride concentration. In order to mimic conditions found *in vivo* $Cl^-$ concentrations at *ca* 140 mM (extra-cellular fluids) and *ca* 2-4 mM (intra-cellular fluids) were used.

The concentration of complex **1** was kept constant at 50 μM in all UV/Vis titrations. Due to solubility problems under these conditions, the chloride concentration was kept below 70 mM. UV/Vis spectroscopy shows that the molar absortivities (ε)) of all bands increase with decreasing ionic strength (I) (Fig. 4a). At equilibrium, the ratio $\varepsilon_{315}/\varepsilon_{358}$ is a good indicator of the evolution of **1** towards **1'** (Fig. 4b). It can also be seen in the same figure that the aquation of complex **1** is complete after *ca* 15 minutes, at a 100 μM $Cl^-$ concentration.

In contrast to the UV/Vis titrations, $^1$H-NMR experiments in $D_2O$ at 37°C [51] allowed the quantification of forms **1** and **1'**, resulting in a correlation between chloride concentration and % aquation. A good correlation was obtained for a second order exponential decay model (Fig. 5). Data analysis suggests that under extra cellular conditions the aqua complex **1'** is present at less than 5 %, however at intra cellular chloride levels it is present at 15- 30%.

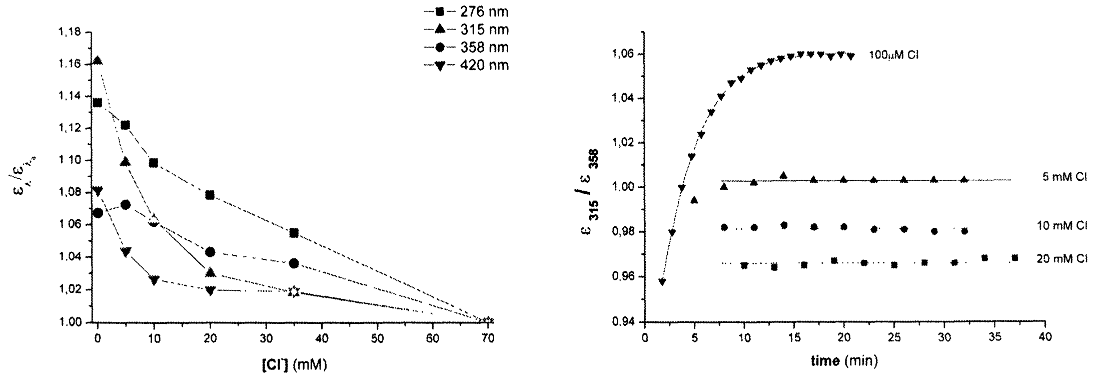

a) Impact of $[Cl^-]$ upon ε: relative intensities for all $\lambda_{max}$ were obtained by comparison with ε values for the solution at $[Cl^-]$=70 mM

b) Evolution of $\varepsilon_{315} / \varepsilon_{358}$ with time and $[Cl^-]$: the increase in the ordinate at equilibrium indicates a higher content of the aquated form **1'**.

**Fig. 4.** Study of the stability of complex **1** with variable chloride content by analysis of its $\varepsilon_{max}$ evolution (Tris buffer 5 mM, 37°C, pH 7.4).

### DNA binding studies

***UV/Visible titrations:*** The intrinsic association constants $K_a$ for the interaction of the ruthenium(II)/dppz complexes **1**, **2** and **3** with CT-DNA were calculated from the UV/Vis titration data, using the following equation:

$$\frac{[DNA]}{\varepsilon_f - \varepsilon_a} = \frac{[DNA]}{\varepsilon_f - \varepsilon_b} + \frac{1}{K_a \times (\varepsilon_f - \varepsilon_a)}$$

where $\varepsilon_a = A_{obs}$ / [complex] and $\varepsilon_f$ and $\varepsilon_b$ correspond to the molar absorptivities of the free and fully bound forms of the complex, respectively. The $[DNA]/(\varepsilon_f-\varepsilon_a)$ ratio was plotted against [DNA] and the constants $K_a$ were obtained from the ratio of the slope to the intercept. [7,52-55]. The data were fitted by a least-squares method ($R^2 \geq 0.999$). $K_a$ values were reproducible for all the complexes and the results are summarised in Table 2.

The calculated $K_a$ values for complexes **1** and **2** are of the same order of magnitude as other DNA intercalating Ru(II)/polypyridyl complexes [2,5,7,13, 33a,56]. Upon titration with CT-DNA the complexes show significant hypochromicity. A red shift of 8 nm is seen for complex **2** while for complex **1** this last effect was difficult to measure due to the absence of a well defined band (see Table

2 and Fig. 3). As the MLCT transition has been assigned to the dppz ligand [26] the hypochromicity can be associated with dppz intercalation into the DNA helix [5,57,58].

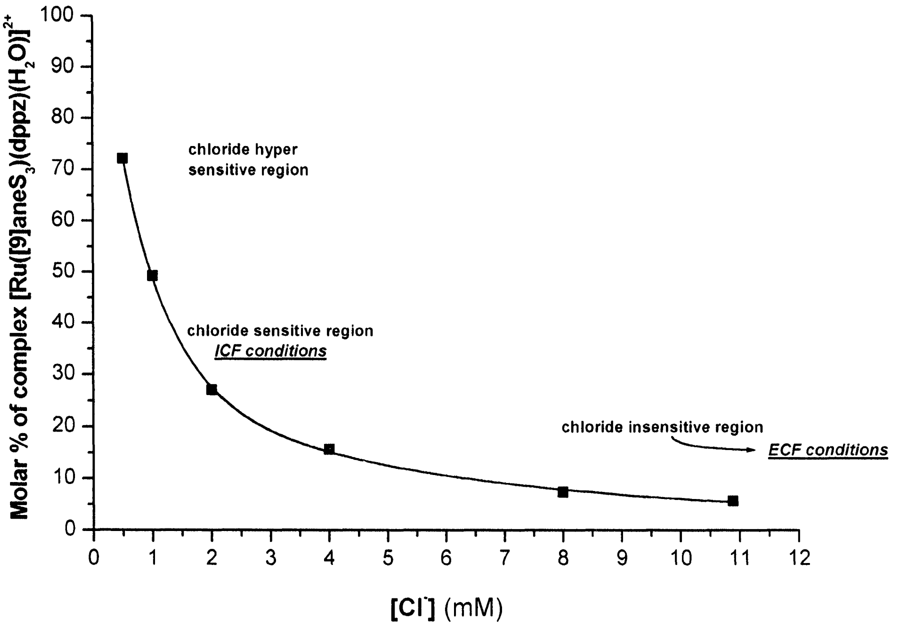

**Fig. 5.** $^1$H-NMR determination of the presence of $[Ru([9]aneS_3)(dppz)(D_2O)]^{2+}$ ($D_2O$; 37°C; sample concentrations from 5.44 mM, to 250 μM; chloride content equals twice the complex concentration).

**Table 2.** UV/Vis DNA titration results

| Complex | $K_a$ _ $10^6$ | Hypochromism / % | red shift /nm |
|---|---|---|---|
| $[Ru([9]aneS_3)(dppz)(H_2O)]^{2+}$ **1'** | 3-5 | 25.2 | [a] |
| $[Ru([12]aneS_4)(dppz)]^{2+}$ **2** | 2-5 | ≈ 24 | 8 |
| $[Ru(bpy)_2(dppz)]^{2+}$ **3** | 2-4 | ≈ 9 [b,c] | [b,c] |
| $[Ru(phen)_2(dppz)]^{2+}$ [d] | 5.1 | 40 [d] | 8 |
| $[Ru(NH_3)_4(dppz)]^{2+}$ [e] | ≈ 0.13 | 13,6 | 0 |
| $[Rh([12]aneS_4)(phi)]^{3+}$ [f] | > 1 | 33 | 9 |
| Ethidium bromide [g] | 1.4 | --- | --- |

[a)] not determined (MLCT shoulder); [b)] this study; [c)] Ref. [1]; [d)] Ref. [6]: refered to double-hump intraligand transition;[e)] Ref. [33a]; [f)] Ref. [10]; [g)] typical intercalator (non-metallo)

In the crystal structure of complex **1** [22] two independent $[Ru([9]aneS_3)(dppz)Cl]^+$ cation chains are assembled in a π-stacking double-helix arrangement with a minimum dppz-dppz inter-planar distance of 3.35 Å. This is analogous to the distance between nucleobases in DNA suggesting that in solution a similar arrangement can be adopted in presence of DNA.

***Steady-state luminescence studies***: The interaction of complexes **1** and **2** with DNA was also studied *via* their luminescence. Results (summarised in Table 3) indicate the binding characteristics of the excited-state Ru(II) complexes. Complex **3** has a strong "light switch" effect and was used to check the emission experiments. As for other Ru(II)-polypyridyl complexes the lower energy excited state is of a MLCT type (Ru (*d*) → L(π*) ) with a triplet emission at 610 nm [26].

**Table 3.** Absorption and emission of complexes **1** and **2** with and without DNA.

| Complexes | Emission * | | | | | | | |
|---|---|---|---|---|---|---|---|---|
| | λ (nm) | | I (×10³) | | Area (×10⁵) | | I/Io | A/Ao |
| | Ru | Ru+DNA | Ru | Ru+DNA | Ru | Ru+DNA | | |
| **1** | 499<br>— | 495<br>614 | ≈2<br>0.33 | ≈12<br>5.37 | 3.4<br>— | 13.0<br>6.5 | ≈ 6<br>16.3 | 3.8<br>— |
| **2** | 569 | 540 | 10.0 | 16.0 | 9.9 | 26.1 | 1.6 | 2.6 |

* 6 μM complex, Ru/DNA 1:30, in 5 mM Tris and 50 mM NaCl, at 23°C. Excitation was carried out at each of the MLCT peaks. *A* represents the area under the band and *I* the intensity of the emission.

For excitation at the MLCT wavelength, complex **1** shows a similar pattern to complex **3**, *ie*, no emission near 610 nm and a significant enhancement of the emission intensity ($\lambda_{max}$ = 614 nm, $I/I_0 \cong 16\times$) after DNA addition (Fig. 6) indicating a strong intercalation. Additionally, a second band at *ca* 500 nm also shows an intensity increase upon DNA addition ($A/A_0 = 3.8\times$). If excitation is carried out at 356 nm there is a smaller increase in emission in the presence of DNA ($\lambda_{max}$= 608 nm, $I/I_0 \cong 10\times$) and the band near 500 nm is replaced by two new bands at 423 and 461 nm. Taking into account that the excitation was carried out at the characteristic dppz "double-humped" transition ($\pi \rightarrow \pi^*$) these two new bands are probably ligand centred.

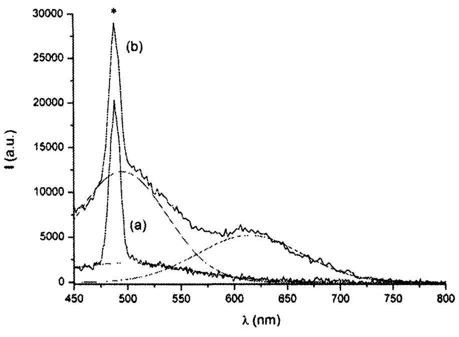

**Fig. 6.** $[Ru([9]aneS_3)(dppz)Cl]^+$ **1** luminescence and deconvoluted spectra: (**a**) 6 μM complex in 5 mM Tris and 50 mM NaCl, under air saturation; (**b**) 1:30 Ru/DNA, keeping all other conditions constant (* Raman band).

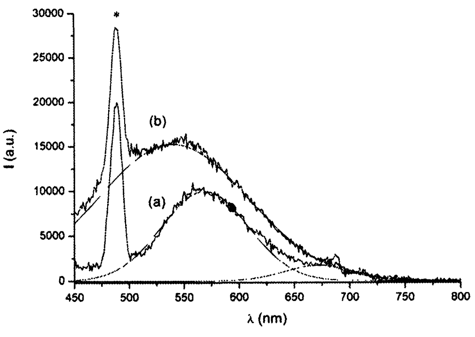

**Fig. 7.** $[Ru([12]aneS_4)(dppz)]^{2+}$ **2** luminescence and deconvoluted spectra: (**a**) 6 μM complex in 5 mM Tris and 50 mM NaCl, under air saturation; (**b**) 1:30 Ru/DNA keeping all other conditions constant (* Raman band). The small band at 670 nm was introduced to obtain a better deconvolution.

Spectral deconvolution of the emission spectrum for complex **2** indicates a band at 569nm and a very low intensity band centred at 672 nm. After DNA addition the first band shifts to 540 nm ($I/I_0 \cong 1.6\times$, $A/A_0 = 2.6\times$). DNA addition also results in a significant MLCT red shift (408 to 416 nm) and in a remarkable blue shift for the corresponding emission (Fig. 7). Considering the magnitude of these shifts, commonly correlated with the strength of the complex intercalation with DNA, this may indicate a strong interaction.

Complex **2** emits even without DNA addition, which is unusual for these type of dppz complexes [59]. Furthermore, the peak is centred at more energetic wavelengths than normally expected. This may be partially understood considering data for Ru(II)-homoleptic complexes with phenanthroline derivatives with bulky groups. These groups diminish the emission wavelength [36] via steric clashes and by geometric distortion. As distortion is seen in the crystal structure of complex **2** ($S_{ax}$-Ru-$S_{ax}$ *ca* 162°) this may explain the appearance of the lower wavelength emission. However more detailed studies are needed to clarify the emission spectra of complex **2** and to fully understand the influence of the macrocycle-type on the emission properties of the Ru(II)-polypiridyl fragment.

***Thermal denaturation studies:*** The thermal conversion of the DNA helix into single strands is characterised by $T_m$ and $\Delta T_m$. An increase in $T_m$ is normally observed for DNA in the presence of intercalators due to the stabilisation of the double helix *via* stacking of planar ligands into the nucleobases [33a,60]. For DNA, and DNA plus the Ru(II)/dppz complexes, melting was followed by UV/Vis spectroscopy. The changes in absorbance with increasing temperature, at 260 nm, were recorded (*See experimental section*). The results are summarised in Table 4. Melting curves were biphasic as expected from similar studies of the thermal denaturation of DNA [60,61].

**Table 4.** Thermal denaturation studies for CT-DNA and CT-DNA/Ru(II)-dppz complexes.

| Compound | $T_m$ (°C) | $\Delta T_m$(°C) |
|---|---|---|
| DNA | 78 [a,b] | --- |
| DNA / [Ru([9]aneS$_3$)(dppz)Cl]$^+$ (10:1) | 91 [a] | +13.0 |
| DNA / [Ru([9]aneS$_3$)(dppz)Cl]$^+$ (10:1) | 87 [b] | +9.0 |
| DNA / [Ru([9]aneS$_3$)(dppz)Cl]$^+$ (20:1) | 90.5 [a] | +12.5 |
| DNA / [Ru([12]aneS$_4$)(dppz)]$^{2+}$ (20:1) | 84.5 [a] | +6.5 |
| DNA / [Ru([12]aneS$_4$)(dppz)]$^{2+}$ (20:1) | 87 [b] | +9.0 |
| DNA / [Ru([9]aneS$_3$)(phen)Cl]$^+$ (5:1) | 81 [a] | +3.0 |

[a)] [DNA] = 125 μM: [b)] [DNA] = 67.5 μM

As can be seen from Table 4 both dppz complexes increased the melting temperature of DNA. Complex **1** showed higher $T_m$ and $\Delta T_m$ values than complex **2**. Under the experimental conditions, complex **1** exists in solution as the aquated form, which bears the same electric charge as **2**. It appears, therefore, that complex **1** is a somewhat better intercalator.

The result for the [Ru([9]aneS$_3$)(phen)Cl]$^+$ complex indicates that complexes **1'** and **2** are both better intercalators. Our data also show that the DNA stabilisation produced by the interaction of complexes **1'** and **2** ($\Delta T_m$) is comparable with data observed for other dppz complexes, for example $\Delta T_m$ = +9.1 °C for [Ru(phen)$_2$dppz]$^{2+}$ [33].

### Concluding remarks

Studies of the influence of non-polypyridyl ancillary ligands on the intercalating abilities of dypyridophenazine Ru(II)-complexes, are still scarce. The results presented here represent the first study of polythioether-Ru(II)-dppz complexes with DNA. The binding constants of the new complexes, [Ru([9]aneS$_3$)dppz(H$_2$O)]$^{2+}$ **1'** and [Ru([12]aneS$_4$)dppz]$^{2+}$ **2**, indicate that they behave as good DNA metallo-intercalators. Thermal denaturation, UV/Vis spectroscopic titrations and steady-state luminescence studies all suggest that intercalation is taking place. The magnitude of the DNA-binding constants is also consistent with the structural characteristics of the complexes. Both the [9]aneS$_3$ and [12]aneS$_4$ macrocyclic rings are smaller than the polypyridyls commonly used in this type of study and thus do not hinder intercalation of dppz in the DNA helix.

The apparent stronger intercalative capacity of the [9]aneS$_3$ complex (seen for the luminescence and thermal denaturation studies) may be due to its smaller overall volume when compared to the [12]aneS$_4$ complex allowing closer approach to the DNA. The labile Cl ligand also allows aquation leading to further reduction in volume. As for the specificity of the two complexes; no direct evidence is available, however for the [9]aneS$_3$ complex, the approach of the axial sulfur to the axial plane might permit VDW interactions between the nucleobases and the macrocycle CH$_2$ groups. Furthermore, the possibility, in the same complex, of exchanging the Cl ligand for a bound water molecule may allow a greater number of specific interactions between DNA and the [9]aneS$_3$ metal complex when compared to the [12]aneS$_4$ analogue.

**Acknowledgments**

The authors acknowledge financial support from the Fundação para a Ciência e Tecnologia (FCT) and PRAXIS XXI (PRAXIS/PCEX/C/QUI/122/96). J. M. also acknowledges the FCT for a PhD grant. V. Félix thanks FCT and the British Council, for travel grants. Professor F. Pina (UNL) is thanked for help with the luminescence studies, EPSRC (UK) and the University of Reading for the

Image Plate System and Mr. A. W. Johans for his assistance with the crystallography. We are also grateful to Prof. M.G. Santana-Marques (mass spectra) and to Prof. F. M. L. Amado for helpful discussions.

**References**

1. A. E. Friedman, J.-C. Chambron, J.-P. Sauvage, N. J. Turro, J. K. Barton, *J. Am. Chem. Soc.*, 1990, **112**, 4960
2. R. E. Holmlin, E. D. A. Stemp, J. K. Barton, *Inorg. Chem.*, 1998, **37**, 29
3. C. Turro, S. H. Bossmann, Y. Jenkins, J. K. Barton, N. J. Turro, *J. Am. Chem. Soc.*, 1995, **117**, 9026
4. E. J. C. Olson, D. Hu, A. Hormann, A. M. Jonkman, M. R. Arkin, E. D. A. Stemp, J. K. Barton, P. F. Barbara, *J. Am. Chem. Soc.*, 1997, **119**, 11458
5. X.-H. Zou, B.-H. Ye, H. Li, Q.-L. Zhang, H. Chao, J.-G. Liu, L.-N. Ji, X.-Y. Li, *J. Biol. Inorg. Chem.*, 2001, **6**, 143
6. C. Hiort, P. Lincoln, B. Nordén, *J. Am. Chem. Soc*, 1993, **115**, 3448
7. A. M. Pyle, J. P. Rehmann, R. Meshoyrer, C. V. Kumar, N. J. Turro, J. K. Barton, *J. Am. Chem. Soc.*, 1989, **111**, 3051
8. R. M. Hartshorn, J. K. Barton, *J. Am. Chem. Soc.*, 1992, **114**, 5919
9. D. L. Carlson, D. H. Huchital, E. J. Mantilla, R. D. Sheardy, W. R. Murphy Jr, *J. Am. Chem. Soc.*, 1993, **115**, 6424
10. H. Krotz, L. Y. Kuo, T. P. Shields, J. K. Barton, *J. Am.Chem. Soc.*, 1993, **115**, 3877
11. C. Moucheron, A. K. de Mesmaeker, J. M. Kelly, *J. Photochem. Photobiol. B*, 1997, **40**, 91
12. J.-Z. Wu, B.-H. Ye, L. Wang, L.-N. Ji, J.-Y. Zhou, R.-H. Li, J.-Y. Zhou, *J. Chem. Soc., Dalton Trans.*, 1997, 1395
13. K. E. Erkkila, D. T. Odom, J. K. Barton, *Chem. Rev.*, 1999, **99**(9), 2777
14. A. M. Pyle, E. C. Long, J. K. Barton, *J. Am. Chem. Soc.*, 1989, **111**, 4520
15. A. M. Pyle, T. Morii, J. K. Barton, *J. Am. Chem. Soc.*, 1990, **112**, 9432
16. A. Sitlani, E. C. Long, A. M. Pyle, J. K. Barton, *J. Am. Chem. Soc.*, 1992, **114**, 2303
17. D. Campisi, T. Morii, J. K. Barton, *Biochem.*, 1994, **33**, 4130
18. J. G. Collins, T. P. Shields, J. K. Barton, *J. Am. Chem. Soc.*, 1994, **116**, 9840
19. A. Krotz, L. Y. Kuo, J. K. Barton, *Inorg. Chem.*, 1993, **32**, 5963
20. B. P. Hudson, J. K. Barton, *J. Am. Chem. Soc.*, 1998, **120**, 6877
21. C. L. Kielkopf, K. E. Erkkila, B. P. Hudson, J. K. Barton, D. C. Rees, *Nature Structural Biology*, 2000, **7**, 117
22. J. Madureira, T. M. Santos, B. J. Goodfellow, M. Lucena, J. P. de Jesus, M. G. Santana-Marques, M. G. B. Drew, V. Félix, *J. Chem. Soc., Dalton Trans.*, 2000, 4422
23. J. K Barton, E. Lolis, *J. Am. Chem. Soc.*, 1985, **107**, 708
24. a) SHELXS-86, G. M. Sheldrick, *Acta Crystallogr.*, 1998, **21**, 916; b) G. M. Sheldrick, SHELX-97, University of Gottingen, 1997
25. J. E. Dickeson and L. A. Summers, *Aust. J. Chem.*, 1970, **23**, 1023
26. E. Amouyal, A. Homsi, J.-C. Chambron, J.-P. Sauvage, *J. Chem. Soc., Dalton Trans.*, 1990, 1841
27. C. Landgrafe and W. S. Sheldrick, *J. Chem. Soc., Dalton Trans.*, 1994, 1885
28. T. M. Santos, B. J. Goodfellow, J. Madureira, J. P. de Jesus, V. Félix, M. G. B. Drew, *New J. Chem.*, 1999, **23**, 1015
29. a) G. Felsenfeld, S. Z. J. Hirschman, *J. Mol. Biol.*, 1965, **13**, 407; b) M. E. Reichmann, S. A. Rice, C. A. Thomas, P. Doty, *J. Am. Chem. Soc.*, 1954, **76**, 3047
30. J. Marmur, *J. Mol. Biol.*, 1961, **3**, 208
31. J. Fees, W. Kaim, M. Moscherosch, W. Matheis, J. Klíma, M. Krej_ík, S. Záli_, *Inorg. Chem.*, 1993, **32**, 166
32. B. J. Goodfellow, V. Félix, S. Pacheco, J. P. de Jesus, M. G. B. Drew, *Polyhedron*, 1997, **16**, 393
33. a) R. B. Nair, E. S. Teng, S. L. Kirkland and C. J. Murphy, *Inorg. Chem.*, 1998, **37**, 139; b) J. M. Kelly, A. B. Tossi, D. J. McConnell, C. Ohvigin, *Nucleic Acid Research*, 1985, **13**, 6017
34. M. G. Santana-Marques, F. M. L. Amado, A. J. Ferrer Correia, M. Lucena, J. Madureira, B. J. Goodfellow, V. Félix, T. M. Santos, *J. Mass Spectrom.*, 2001, **36**, *in press*
35. L. Antonov and D. Nedeltcheva, *Chem. Soc. Rev.*, 2000, **29**, 217
36. A. Juris, V. Balzani, F. Barigelletti, S. Campagna, P. Belser, A. Von Zelewsky, *Coord. Chem. Rev.*, 1988, **84**, 85
37. B. J. Goodfellow, V. Félix, S. Pacheco, J. P. de Jesus, M. G. B. Drew, *Polyhedron*, 1997, **16**, 3293
38. F. M. L. Amado, C. M. Barros , M. G. Santana-Marques, P. M. Domingues, A. J. Ferrer-Correia, J. Madureira, T . M. Santos, V. Félix, *48$^{th}$ Conference* ASMS, 2000, Palm Springs, USA
39. S. Roche, H. Adams, S. E. Spey, J. A. Thomas, *Inorg. Chem.*, 2000, **39**, 2385
40. M. N. Ackermann, L. V. Interrante, *Inorg. Chem.*, 1984, **23**, 3904
41. C.-K. Poon, S.-S. Kwong, C.-M. Che, Y.-P. Kan, *J. Chem. Soc., Dalton Trans.*, 1982, 1457

42. T.-F. Lai, C.-K. Poon, *J. Chem. Soc., Dalton Trans.*, 1982, 1465
43. S. C. Rawle, S. R. Cooper, *Chem. Commun.*, 1987, 308
44. S. C. Rawle, T. J. Sewell, S. R. Cooper, *Inorg. Chem.*, 1987, **26**, 3769
45. M. N. Bell, A. J. Blake, A. J. Holder, T. I. Hyde, M. Schröder, *J. Chem.Soc., Dalton Trans.*, 1990, 3841
46. N. W. Alcock, J. C. Cannadine, G. R. Clark, A. F. Hill, *J. Chem.Soc., Dalton Trans.*, 1993, 1131
47. W.-C. Cheng, W.-Y. Yu, K.-K. Cheung, C.-M. Che, *J. Chem. Soc., Dalton Trans.*, 1994, 57
48. J. Fees, M. Ketterle, A. Klein, J. Fiedler, W. Kaim, *J. Chem. Soc., Dalton Trans.*, 1999, 2595
49. L. Spek, PLATON, a *Multipurpose Crystallographic Tool,* Utrecht University, Utrecht, The Netherlands, 1999
50. Crystal data: $C_{26}H_{26}Cl_2NRuS_4$, $M$=649.72, Triclinic, space group $P\bar{1}$ ($a$=7.613(11), $b$=10.371(21$_3$), $c$=21.676 (31) Å, $\alpha$=79.01(1), $\beta$ =87.56(1), $\gamma$= 81.08(1)) $V$ = 1660 Å$^3$, $Z$ = 2, $D_{calcd}$ = 1.390 Mg m$^{-3}$
51. B. J. Goodfellow, J. Madureira, T. M. Santos, V. Félix , unpublished results
52. S. Mhadven, M. Paalaliandavar, *Inorg. Chim. Acta*, 1997, **254**, 291
53. S. J. Heater, M. W. Carrano, D. Rains, R. B. Walter, D. Ji, Q. Yan, R. S. Czernuszewicz and C. J. Carrano, *Inorg Chem*, 2000, **39(17),** 3881
54. S. Mahadven, M. Palaniandavar, *Inorg. Chem.*, 1998, **37(4)**, 693
55. A. Wolfe, G. H. Shimer Jr, T. Meehan, *Biochem.*, 1987, **26**, 6392
56. T. M. Santos, F. M. L. Amado, V. Félix, B. J. Goodfellow, J. P. de Jesus and M. G. B. Drew, *4$^{th}$ Biological Inorg. Chem. Conference*, Book of Abstracts, MM-61, 20-25 July, 1998, Seville, Spain
57. S. Mahadven, M. Palaniandavar, *Inorg. Chem.,* 1998, **37(3)**, 563
58. J. K. Barton, A. T. Danishefsky, J. M. Goldberg, *J. Am. Chem. Soc.*, 1984, **106**, 2172
59. Ref. **13** *in* ref. [33a]
60. Y. Kan, G. B. Schuster, *J. Am. Chem. Soc.*, 1999, **121(50),** 11607
61. S. Satyanarayana, J. C. Dabrowiak, J. B. Chaires, *Biochem.*, 1993, **32(19),** 2573

**Received: May 28 2001 – Accepted: June 18, 2001 – Accepted in publishable format: June 19, 2001**

